# Future trends of display technology: micro-LEDs toward transparent, free-form, and near-eye displays

**DOI:** 10.1038/s41377-025-02027-1

**Published:** 2025-09-22

**Authors:** Tae Soo Kim, Jung-El Ryu, Jinhong Park, Rih-Jia Liu, Joonghoon Choi, Jeehwan Kim, Young Joon Hong, Dong-Hwan Kim, Jiho Shin

**Affiliations:** 1 Artie McFerrin Department of Chemical Engineering, Texas A&M University, College Station, TX 77843 USA; 2 Department of Mechanical Engineering, Massachusetts Institute of Technology, Cambridge, MA 02139 USA; 3 Research Laboratory of Electronics, Massachusetts Institute of Technology, Cambridge, MA 02139 USA; 4 Department of Nano Science and Technology, Sungkyunkwan University, Suwon, 16419 Republic of Korea; 5 SKKU Advanced Institute of Nanotechnology, Sungkyunkwan University, Suwon, 16419 Republic of Korea; 6 School of Chemical Engineering, Sungkyunkwan University, Suwon, 16419 Republic of Korea

**Keywords:** Organic LEDs, Displays, Inorganic LEDs

## Abstract

Displays are one of the most indispensable electronic devices used in our daily lives. Over the past decades, display technology has evolved relentlessly, driven by innovation in materials, structures, and manufacturing processes that have enabled higher image quality, larger screen size, slimmer form factor, and novel functionalities. The display market is currently dominated by liquid crystal displays (LCDs) and organic light-emitting diode (OLED) displays, but significant investment and research efforts are being directed toward emerging self-emissive display technologies, such as micro-light-emitting diodes (micro-LEDs), as well as unconventional applications such as transparent, deformable, and near-eye displays. This review article begins with a historical background of self-emissive display technology and an overview of the recent advances in organic-, quantum dot-, perovskite-, and micro-LED displays. We then critically review the current state of micro-LED technology, including its size-dependent performance issues, different types of mass transfer technologies, backplane interconnection techniques, methods for detection/repair of defective pixels, and emerging display applications, including transparent, deformable, and virtual and augmented reality (VR/AR) displays.

## Abstract (web summary)

Micro-LEDs will lead next-generation display technology such as transparent, deformable and near-eye displays, enabling a wide range of applications with superior performance.

## Graphical abstract

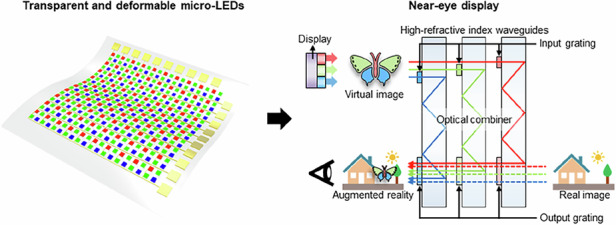

## Evolution of self-emissive display technology

**Fig. 1:**
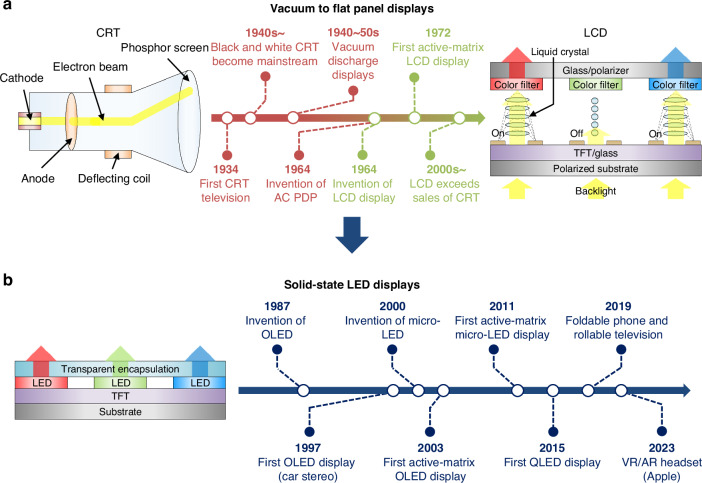
**Evolution of self-emissive display technology.** **a** Device structures of CRT and LCD, and timelines of the transition from vacuum displays to flat panel displays. **b** Device structures and timelines of LED displays

Displays play a vital role in the modern digital age by allowing fast and efficient distribution of knowledge through visual data, which accounts for 80% of the sensory information that humans collect daily^1,2^. Since the commercialization of black-and-white cathode ray tube (CRT) televisions in 1934, innovation in display materials and architectures has enabled more vibrant and realistic images via improved image resolution, contrast ratio, brightness, and response speed^1,3^. CRT displays were the first widely used display technology, finding applications in televisions, computer monitors, and other information delivery devices^4–6^. They worked by firing an electron beam from a cathode towards a phosphor-coated screen, which required bulky/heavy glass vacuum tubes and led to high power consumption (Fig. 1a, left). The vacuum-based display technology later evolved into plasma display panels (PDPs), which adopted a flat-panel design suitable for larger screen sizes. However, PDPs still faced challenges such as high power usage, the need for cooling fans for heat dissipation, susceptibility to screen burn-in, and relatively short lifetime^7^.

By the early 2000s, liquid crystal displays (LCDs) took over the market thanks to their significantly lower power consumption and lighter weight^8^. They contain a layer of liquid crystals sandwiched between two polarized substrates (Fig. 1a, right), which undergoes alignment and rotation upon applying current (via thin-film transistors (TFTs)), thereby modulating the transmitted light intensity. Because liquid crystal molecules behave as partially ordered fluids, their reorientation under an electric field is inherently slow. This leads to typical on-off response times of 5–25 ms. To address this limitation, advanced driving techniques such as overdrive and undershoot voltage schemes have been developed, which can shorten gray-to-gray response times to approximately 1 ms on average^9,10^. Additionally, they exhibit a limited contrast ratio, primarily due to their inability to achieve true black levels, since the backlight continuously emits some residual light. To mitigate this limitation, mini-LED backlight technology has been introduced to enable precise local dimming control, which effectively suppresses residual luminance in dark regions and substantially improves the contrast ratio of LCDs^11^. Nevertheless, due to inherent light leakage through the liquid crystal layer and the inability to completely turn off the backlight at the pixel level, mini-LED backlit LCDs still face fundamental limitations in delivering contrast ratio compared to those of self-emissive displays.

A major advancement in display technology has been the transition from vacuum- and fluid-based platforms to solid-state devices utilizing microelectronic fabrication processes. Following the introduction of the first low-voltage organic light-emitting diode (OLED) by C.W. Tang and VanSlyke in 1987^12^, OLED displays became the most popular in the late 2000s, due to their superior contrast ratio, enabled by using self-emitting red, green, and blue OLED pixels that can be individually controlled (Fig. 1b)^8,13^. OLED displays are also slimmer and lighter than LCDs because they utilize thin layers of organic semiconductors and do not require a separate backlight unit^14^. Quantum dot light-emitting diode (QLED) displays were another type of solid-state LED display first introduced in 2015. Despite their name, QLED displays fundamentally remain LCDs that use quantum dots (QDs) as color converters. However, this approach improved color accuracy and brightness compared to traditional LCDs equipped with color filters. Nonetheless, OLED and QLED display technologies have several limitations: OLEDs suffer from relatively short lifespans, susceptibility to high temperatures and humidity, and limited maximum brightness levels (<4000 nits)^15^, while QLED displays exhibit inherently low contrast ratios.

To overcome these limitations, the display community has been searching for the next-generation display technology, the most prominent of which today is micro-light-emitting diodes (micro-LEDs)^16–19^. Jiang et al. were the first to demonstrate micro-LED arrays capable of passive matrix addressing, laying the foundation for self-emissive microdisplays^20^. Micro-LED displays consist of inorganic-based self-emitting red, green, and blue LEDs with microscale dimensions, which offer higher brightness, lower power consumption, faster response speed, better durability, and longer lifetime compared to OLED and QLED displays^21^. Micro-LED displays became commercially available in the early 2010s, but they are still incredibly expensive today due to high manufacturing costs, which is largely attributed to the low maturity of mass transfer techniques needed to assemble red, green, and blue micro-LED chips on the display panel^22^. QDs and perovskites are also being explored as emissive materials, but their development has been less successful compared to that of micro-LEDs. We summarize the distinct characteristics and fabrication processes of display materials in use or under development in the ensuing section.

## Display materials, structures, and fabrication processes

**Fig. 2:**
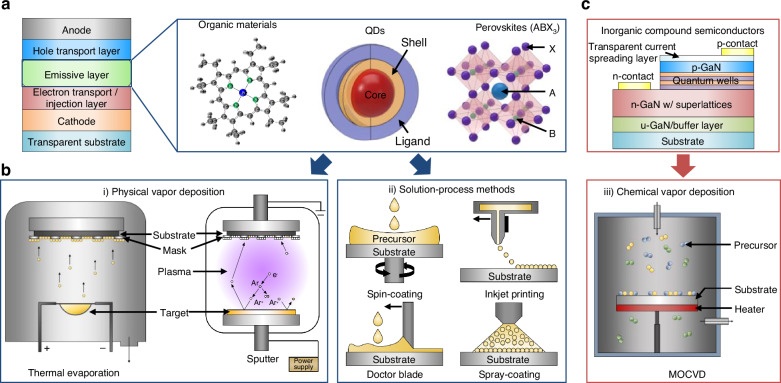
**Self-emissive materials for LEDs.** **a** Structures and emissive materials used for OLEDs, QD-based LEDs, and PeLEDs. **b** Deposition methods for emissive materials: Organic materials, QDs, perovskites, and inorganic compound semiconductors. **c** Representative structure of micro-LEDs

The four most widely explored self-emissive materials for display applications today include OLEDs, QDs, perovskites, and micro-LEDs. QDs are used as color converters in commercial QLED displays, but they can also serve as a direct emitter (hereon, ‘QD-LED’). The basic architectures of OLEDs, QD-LEDs, and perovskite-LEDs are nearly identical, consisting of an n-type electron-injection layer and p-type hole-injection layer, but differing in the emissive layer composition (Fig. 2a). In contrast, micro-LEDs have a more sophisticated emissive layer structure that includes a multiple-quantum well (MQW) structure (Fig. 2c). Below, the unique material properties, device constructs, and fabrication techniques for the four types of self-emissive displays are discussed.

OLEDs, the most widely used display material in mobile devices today, consist of organic-based emissive and charge transport layers that are obtained via physical vapor deposition techniques, such as thermal evaporation and sputtering (Fig. 2a, b, i). Upon applied voltage, both electrons and holes are injected into the emissive layer, generating electroluminescent light with characteristic wavelengths depending on the emissive layer composition. Emissive materials of OLEDs have evolved over time, from fluorescent OLEDs in the early days to phosphorescent OLEDs and thermally activated delayed fluorescent (TADF) OLEDs used today. Both phosphorescent and TADF OLEDs can harness triplet excitons in addition to singlet excitons, which allows them to achieve theoretical internal quantum efficiencies (IQEs) of 100%. This is enabled by employing a host-guest system, wherein the “host” material with defined highest occupied molecular orbital (HOMO) and lowest unoccupied molecular orbital (LUMO) energy levels is doped with small quantities of a “guest” material with different energy levels. This approach facilitates efficient exciton transfer and light emission with finely tunable wavelengths^23–25^.

OLED displays offer several advantages over LCDs, the most important of which is the ability to achieve perfect black levels and an almost infinite contrast ratio, owing to the use of self-emitting RGB subpixels that can be turned on/off individually. They also feature a wider viewing angle, reduced thickness, and faster response time^26^. However, OLEDs face challenges such as vulnerability to moisture and heat, material degradation due to electrochemical reactions^27,28^, thermomechanical failures^29,30^, and exciton quenching^31,32^. Moreover, the fine metal masks (FMMs), typically made of invar alloy (FeNi36), that are used to pattern RGB subpixels side-by-side, present significant manufacturing challenges. Specifically, thinning the FMM to enable finer pattern resolution often causes mechanical sagging, thereby hindering large-area processing and complicating precise mechanical alignment. Additionally, improving the efficiency and lifetime of blue OLEDs remains a critical challenge, as the current blue OLEDs exhibit limited external quantum efficiencies (EQEs; <25%) and short operating lifetime (<500 hr) at 1000 nits^33^. Lastly, the moderate brightness of OLED displays (~10^3^ nits) reduces their visibility under high ambient light intensities^8,34^, limiting their application in AR displays.

QDs are semiconductor nanoparticles consisting of an inorganic semiconductor core, a wider bandgap shell, and an organic passivating ligand layer, typically synthesized via colloidal methods. Owing to controlled mono-dispersity and exceptional physical properties, QDs have precisely tunable emission wavelengths across the entire visible spectrum, narrow emission linewidths, and high photoluminescence quantum yield (PLQY)^35,36^. QD-based color converters exhibit high color purity and brightness, allowing QLED displays to have superior image quality and display performance over LCDs that use color filters^37–39^. There are also efforts to integrate them with monochrome (usually blue) micro-LED displays to achieve full-color displays^40,41^. Conventional methods for patterning QD-based color converters include optical lithography using a lift-off process^42^, inkjet printing using digitally controlled nozzles^43^, and transfer printing using PDMS-based elastomeric stamps^44^.

QD-LEDs, using QDs as direct emissive materials, have shown promise as future display technology owing to their high EQEs (38.2%^45^, 29.2%^46^, and 23%^47^ for red, green, and blue emissions, respectively), but their main limitations include low stability and reliability. QD-LEDs typically adopt hybrid heterojunction architectures composed of an inorganic n-type nanoparticle layer and organic p-type hole-transport (or injection) layers, which are fabricated using solution-based film casting techniques such as spin-coating, inkjet printing, doctor blading, and spray-coating (Fig. 2a, b, ii). Hybrid p–n heterojunctions often suffer from performance limitations due to exciton quenching, non-radiative recombination (e.g., Auger recombination^48,49^), and Joule heating^50,51^, arising from sub-optimal charge carrier balance and defects generated due to exposure to humidity/oxygen^52,53^. Moreover, achieving large-area, high-resolution QD-LED displays is hindered by the inherently low uniformity, reproducibility, and scalability of solution-based spin-casting fabrication processes.

Perovskite LEDs (PeLEDs) consist of metal halide perovskites (MHPs), whose A, B, and X sites in the ABX_3_ perovskite crystal structure are occupied by Cs^+^, methylammonium (MA^+^), or formamidinium (FA⁺); Pb^2+^ or Sn2^+^; and Cl^−^, Br^−^, or I^−^, respectively (Fig. 2a). This crystal structure shares similarities with the double heterostructure (DH) or QWs of micro-LEDs, allowing PeLEDs to have a small FWHM and high color purity with modulated emission colors depending on the composition of ABX_3_^54,55^. The bandgap and emission wavelengths of PeLEDs can be easily tuned by adjusting the types and sizes of perovskite nanocrystals (NCs)^56,57^. PeLEDs also have low manufacturing costs thanks to a solution-based fabrication process (Fig. 2b, ii)^58^. The EQEs of PeLEDs have significantly improved over the years, achieving 28.7%^59^, 29.5%^60^, 23.5%^61^ for red, green, and blue LEDs, respectively. However, their limitations include vulnerability to Joule heating^62^, poor uniformity^63^, and toxicity of Pb-based perovskites. A recent study reported solution-processed PeLEDs that maintain high EQEs despite having sub-100 nm pixel size and an unprecedented pixel density of 127,000 pixels per inch (ppi)^64^.

**Fig. 3:**
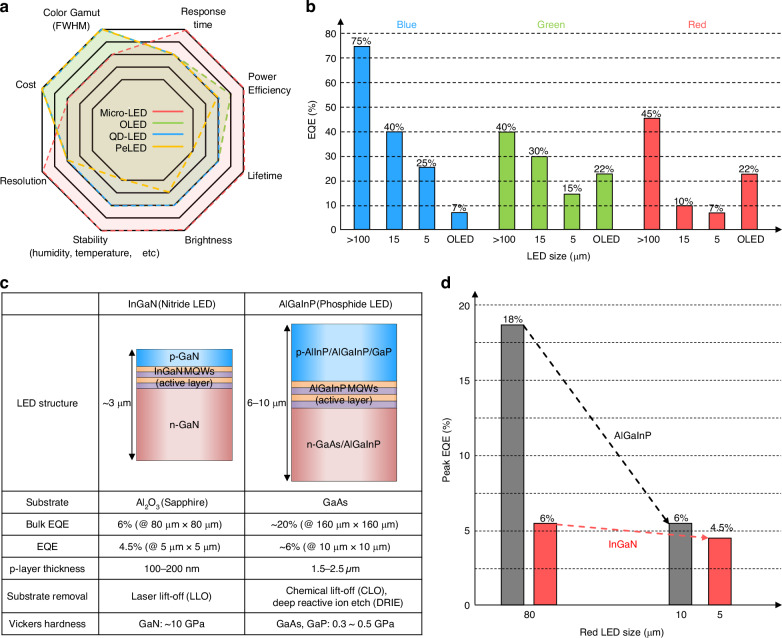
**Size-dependent efficiency loss in micro-LEDs.** **a** Comparison of micro-LEDs and other emissive LEDs. **b** EQE degradation based on micro-LED size. **c** Characteristics of InGaN- and AlGaInP-based red LEDs. **d** EQE variation based on the size of InGaN- and AlGaInP-based red LEDs

Micro-LEDs represent the most promising candidate for next-generation high-performance display applications. They are inorganic semiconductor p–n junction diodes with sub-100 µm size, composed primarily of III–V (e.g., AlGaInP/GaAs) and III–N (e.g., InGaN/GaN) compound semiconductor heterostructures for red and green/blue emissions, respectively. Micro-LEDs are epitaxially grown using metal-organic chemical vapor deposition (MOCVD) or molecular beam epitaxy (MBE) processes, where the controlled injection of precursor gases leads to uniform thin-film deposition at high temperatures (Fig. 2b, iii). The active regions of LEDs consist of multiple-quantum-wells (MQWs) alternating semiconductor layers with varying band gaps, which confine electrons and holes effectively (Fig. 2c). Upon the application of voltage, injected electrons and holes recombine efficiently within MQWs, emitting photons with wavelengths determined by the composition and thickness of the MQWs. This strong carrier confinement promotes efficient radiative recombination and reduces sensitivity to external perturbations, allowing LEDs to achieve superior brightness, contrast ratio, response speed, power efficiency, functional lifetime, and stability in humid/high-temperature environments over OLEDs, QD-LEDs, and PeLEDs, as summarized in the radar plot in Fig. 3a^39,65–70^. For example, the luminance, IQE, and response speed of typical blue InGaN LEDs are known to be >200 lm W^−1^ (corresponding to >10^7^ nits)^71^, nearly 100%^72^, and 1–10 ns^73^, respectively. However, there are currently two major technical challenges—the size-dependent efficiency loss and the low maturity of mass transfer techniques—which are summarized in the following sections.

## Size-dependent efficiency loss of micro-LEDs

The dramatic reduction in power efficiency of micro-LEDs as their lateral dimensions decrease is a major hindrance to the broader adoption of the technology, especially for micro-display applications. Below 20 µm chip size, the EQE decreases sharply due to an increased proportion of surface defects leading to non-radiative recombination at device sidewalls (Fig. 3b)^74^. This is particularly pronounced for red-emitting AlGaInP micro-LEDs, which exhibit higher surface recombination velocities and longer carrier diffusion lengths, intensifying carrier loss at the chip sidewalls^75,76^. Consequently, while InGaN-based blue and green micro-LEDs maintain relatively high EQEs even at reduced dimensions (for example, 40% and 30% for 15 µm chip size, respectively), red micro-LEDs experience a sharper reduction (10% for 15 µm chips). This has prompted research efforts toward improving device structures, optimizing epitaxial growth conditions, and exploring alternative material systems to achieve balanced and efficient full-color displays.

One potential solution that is drawing major attention is the development of InGaN-based red micro-LEDs. Unlike red-emitting AlGaInP LEDs that are typically grown on GaAs substrates, red InGaN LEDs are grown on sapphire substrates and have composition and epitaxial structure similar to those of green and blue InGaN LEDs (see illustration in Fig. 3c). InGaN-based red micro-LEDs are inherently less sensitive to sidewall-related surface non-radiative recombination, allowing them to better retain EQE at smaller chip size (Fig. 3d)^76,77^. Moreover, they hold promise for enabling fully InGaN-based RGB micro-LED displays, which can reduce manufacturing complexity/costs due to the similarity in structure and processing methods (i.e., MOCVD growth, chip singulation, substrate separation, mass transfer, integration/bonding, electrical interconnection, etc.). However, achieving homogeneous high indium content in MQWs needed for red emission remains challenging, as it necessitates lower-temperature epitaxial growth, which typically results in increased lattice mismatch, compromised crystal quality, and reduced luminous efficiency^78–81^.

## Mass transfer technologies

**Fig. 4:**
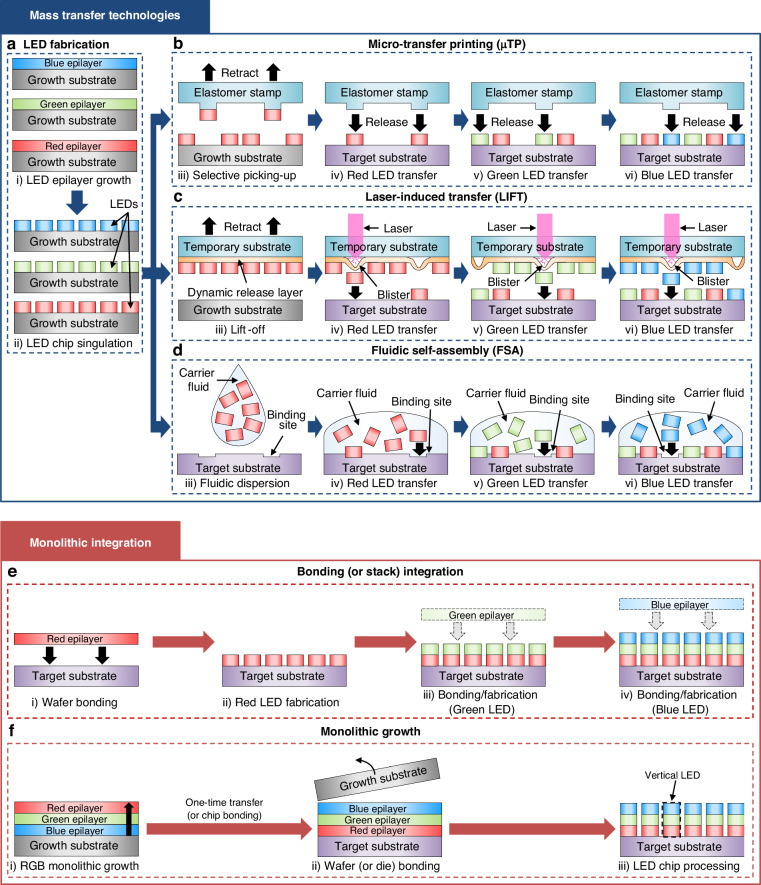
**Mass transfer and monolithic integration technologies.** **a** Schematic illustration of LED fabrication. **b** Schematic illustration of the elastomer stamp transfer process. **c** Schematic illustration of laser-induced transfer. **d** Schematic illustration of fluidic self-assembly. **e** Schematic illustration of bonding (or stack) integration of RGB LED layers. **f** Schematic illustration of the monolithic growth

Another major challenge for micro-LED display technology is the low maturity of mass transfer techniques, which are needed to integrate the fabricated RGB micro-LED chips with the CMOS backplane for active-matrix operation. Mass transfer is a process uniquely required for micro-LED displays, because red, green, and blue micro-LED chips can only be produced on single-crystalline wafers with a similar lattice structure, typically GaAs wafers for AlGaInP-based red LEDs and sapphire wafers for InGaN-based green and blue LEDs (Fig. 4a)^82^. The fabricated chips are then selectively transferred to the target substrate—either the backplane or a temporary substrate—where they are arranged at a larger pixel pitch than they were on the growth substrate. This is crucial for minimizing costs, as commercial micro-LED televisions have millimeter-scale pixel pitch, and distributing tiny micro-LED chips (<100 µm) at larger spacing allows fewer chips to be used to cover the same area.

Despite various efforts, the high costs of equipment, high percentage of defective pixels due to misaligned or missing chips, and the need for rigorous quality control are driving up the manufacturing costs of micro-LED displays compared to OLED and QLED displays. This is amplified by the large number of chips needed to build displays. A 4 K resolution display requires approximately 8 million pixels (or 24 million RGB micro-LED chips), which indicates that even with an extremely low defect rate of 0.01%, ~2400 subpixels will be defective and require an extensive repair process. Therefore, various mass transfer techniques are under development with the goal of achieving a transfer yield and rate of 99.9999% and 100 million units per hour, respectively, to minimize manufacturing costs.

In this section, we introduce the mechanisms and characteristics of the four most widely explored mass transfer approaches: micro-transfer printing, laser-induced forward transfer, fluidic self-assembly, and monolithic integration. Their characteristics and key parameters (chip size, transfer rate, yield, and precision) are also summarized in Table 1.

**Table 1:** Benchmark table comparing key parameters of various mass transfer techniques

	Micro-transfer printing	Laser-induced forward transfer	Fluidic self-assembly	Monolithic integration
Mechanism	Adhesion control	Laser irradiation	Surface tension Fluid dynamics	Vertical stacking
Advantage	High throughput Works for various materials	Non-contact process High throughput	Low cost High throughput	High alignment accuracy High-pixel density
Disadvantage (general)	Yield issues Process complexity	Laser damage High cost	Low precision Complex chip design	High cost Process complexity
Chip size	~5 μm	~50 μm	>20 μm (diameter)	<5 μm
Transfer rate (units/h)	>6.5 M	>100 M	>50 M	N/A
Transfer yield (published)	>99.99%	>90%	99.88%	N/A
Transfer precision (μm)	1.5	1.8	1.25	<<0.1 (No transfer)
Company/institution	X Display Company, KIMM	Uniqarta	eLux SAIT LG Electronics	Ostendo, MIT, JBD, Porotech, Sundiode/Soft-Epi
Ref.	^87,88^	^95^	^98–101^	^104,106–108^

Micro-transfer printing (µTP), also denoted as the ‘pick-and-place’ method, utilizes stamps made of elastomers such as polydimethylsiloxane (PDMS) to “pick” up micro-LED chips from their growth substrate and to “place” them at precisely aligned locations on the target substrate (Fig. 4b). These processes rely on the kinetic control of adhesion, as retracting the stamp at higher/lower speeds increases/reduces the van der Waals (vdW) force between the stamp and substrate, respectively, owing to the viscoelastic behavior of elastomer^83^. Rogers et al. first explored this method to demonstrate monochrome micro-LED displays^84–86^, while X Display Company (XDC), a spin-off of X-Celeprint, recently reported a transfer yield of over 99.9% for their transfer printing process^87–90^. Elastomers with cylindrical shape have been used to demonstrate ‘roll-to-plate transfer’, allowing semi-continuous transfer of Si TFTs and micro-LEDs for building monochrome active-matrix micro-LED displays^91^. Overall, µTP offers high throughput, yield, and versatility (can be used for all materials, including InGaN, AlGaInP, etc.), but it suffers from yield issues and process complexity.

Laser-induced forward transfer (LIFT) relies on controlled irradiation of a laser to selectively release and transfer micro-LED chips^92^. This begins with a laser lift-off (LLO) process that separates fabricated micro-LED chips onto a transparent temporary substrate coated with a dynamic release layer (DRL) (Fig. 4c)^93,94^. Laser irradiation through the bottom side of the temporary substrate causes DRL to vaporize, inducing local expansion and selective transfer of micro-LED chips to the target substrate. Uniqarta introduced massively parallel laser-enabled transfer (MPLET) technology that enables a transfer rate of ~100 M units per hour using a single laser source, diffractive optical element, and laser scanner^95^. The biggest advantage of LIFT over other mass transfer methods is the remarkably high transfer rate with a scan cycle speed of 1–10 kHz. Current limitations include the low alignment accuracy and risks of mechanical damage as the micro-LED chips are released/dropped from long distances (relative to chip dimensions); high costs of equipment and operation; and the unintended release of neighboring chips due to the wide spatial profile of laser beams. To overcome these limitations, in situ bonding techniques are being developed in conjunction with the LIFT process, offering a promising route to simultaneously achieve both transfer and bonding in a single integrated step^95,96^.

Fluidic self-assembly (FSA) involves dispersing the micro-LED chips in a carrier fluid, which then allows them to self-assemble onto the target substrate with specially designed binding sites due to surface tension and fluid dynamics (Fig. 4d)^97^. Different micro-LED chip designs and self-assembly mechanisms have been explored to improve the transfer rate and yield. eLux reported chips with the shape of disks with thin cylindrical posts attached on the opposite side of contact pads^98^, while Samsung Advanced Institute of Technology (SAIT) demonstrated chips with low-vdW-force material with high roughness on one surface and high-vdW-force material with low roughness on the other^99^. LG Electronics reported magnetic-force-assisted dielectrophoretic self-assembly technology (MDSAT) that allows concurrent transfer of red, green, and blue micro-LED chips^100^, while Lee et al. demonstrated the addition of poloxamer to carrier fluid to increase its viscosity, thereby improving liquid-to-chip momentum transfer as well as the transfer yield^101^. The key advantages of FSA include high throughput and low costs of materials/equipment. Their limitations include the risks of chip damage due to collision during the assembly process; complex chip designs required to improve yield and selectivity, especially for ultra-high-resolution displays; and agglomeration or attachment of chips due to van der Waals or electrostatic forces. Nevertheless, FSA holds great potential as a cost-effective and scalable solution for high-throughput micro-LED assembly.

Monolithic integration, also referred to as the ‘vertical stacking’ method, involves stacking ultrathin red, green, and blue LED layers vertically and using top-down fabrication to obtain full-color micro-LEDs (Fig. 4e, f). The LED layers can be stacked in the ascending order of bandgap energy (R-G-B) to minimize the absorption of light emitted by the smaller-bandgap LEDs by the layers above. Monolithic integration offers unique advantages for manufacturing micro-displays with pixel pitch <10 µm, because: (i) the pixel density can be improved three-fold compared to conventional displays with lateral RGB pixel layouts, and (ii) the volume of LED removed during the mesa etching step is minimized due to the high pixel density, thereby reducing materials cost^102^. In their ‘2022 MicroLED report’, Yole Intelligence suggested that an 8 K display using 34 μm × 58 μm chips could cost roughly 24,000 USD in chip costs alone, while vertically stacked RGB micro-LED chips with 5 μm × 5 μm size could reduce the cost to 130 USD. In addition, while mass transfer techniques are not yet optimized for <30 µm chip size, monolithic integration can achieve extremely high alignment accuracy due to the significantly larger size of the transferred object (wafer-sized LEDs) and the reduced frequency of transfer. Following transfer, micro-LEDs can be patterned with high alignment accuracy via photolithography by aligning with respect to the underlying CMOS circuitry. Deep ultraviolet (DUV) systems, for example, routinely offer overlay precision of >50 nm, which is more than sufficient for fabricating 2–3 μm-sized LEDs required for 5000 ppi-class AR displays.

Currently, two approaches to achieving monolithic integration include: (i) repeating wafer bonding, lift-off, and fabrication steps three times (for each of the RGB LED layers), and (ii) epitaxially growing InGaN-based RGB LED structures sequentially. The first approach was demonstrated by Ostendo, who monolithically integrated AlGaInP-based red and InGaN-based green and blue LEDs by using lapping, wet etching, and LLO techniques (Fig. 4e)^103^. Shin et al. reported monolithic integration of AlGaAs-based red and InGaN-based green and blue LEDs using a two-dimensional materials-based layer transfer (2DLT) technique, whereby LED layers grown on wafers coated with 2D materials (graphene, boron nitride) are peeled off using thermal-release tapes^104^. Stacking RGB LED layers (thickness: 1–2 μm each) with polyimide bonding layers enabled a vertically stacked tandem LED structure with ~9 μm total thickness, 4 μm footprint, and 5 μm pixel pitch, which corresponds to ultra-high resolution of 5100 ppi. Jade Bird Display (JBD) also reported vertically stacked micro-LED technology with similar form factor^105^. To achieve complete display, additional work is needed to demonstrate integration of vertical micro-LED chips with CMOS backplane for active-matrix operation (discussed further in the next section).

The monolithic growth method, whereby InGaN-based RGB LEDs are grown sequentially on a single wafer, has been demonstrated by display manufacturers Ostendo^106^, Porotech, and Sundiode/Soft-Epi (Fig. 4f)^107,108^. While this method offers simpler manufacturing process (no wafer bonding and lift-off steps), it has critical limitations such as: (i) the inability to independently control the color and brightness of RGB subpixels; (ii) the inherently low EQE of InGaN-based red LEDs, which is exacerbated by the thermal budget accumulated over the sequential growth process; and (iii) high contact resistance of p-type electrodes due to the diffusion of Mg dopants far across the junction at high sequential growth temperatures. Further efforts are needed to suppress Mg diffusion and ensure reliable performance of tunnel junction-based vertically stacked LEDs^109^. Meanwhile, monolithic growth of nanowire LEDs provides an alternative solution for creating full-color micro-displays with high-pixel density^110–112^. Their inherently small size and geometry are well-suited for ultra-high-pixel density displays, enabling “built-in pixel redundancy” to minimize dead pixels. With careful control over selective area growth conditions, it is possible to monolithically grow red, green, and blue InGaN/GaN nanowire LEDs on a single substrate^110^, thereby simplifying integration.

## Backplane interconnection techniques

Following the mass transfer process, micro-LEDs must be electrically interconnected with a display backplane, which consists of circuits of transistors and capacitors that enable control of brightness and on/off states of individual subpixels. This process is simple for OLED displays, as the RGB subpixels can be directly deposited on the contact pads of the backplane. Micro-LED displays require a different approach because the high growth temperatures of inorganic LEDs can damage the CMOS circuit. Currently, large-scale micro-LED displays utilize flip-chip bonding, while micro-displays rely on wafer bonding. Another method is growth integration, where TFTs are grown on LED wafers and interconnected monolithically. A comparison of these methods is provided in Table 2.

**Table 2:** Benchmark table comparing key parameters of backplane interconnection techniques

Interconnection method	Pixel size (µm)	Pixel pitch (µm)	Resolution	ppi	Ref.
Flip-chip bonding	9	12	320 × 140	N/A (ultraviolet)	^113^
	8	12.8	960 × 540	2000 (blue)	^114^
	12	15	640 × 480	N/A (green, blue)	^115^
	6	8	1920 × 1080	3175 (red, blue)	^116^
Wafer bonding	N/A	2.5	N/A	N/A (red)	^105^
	~5	5	2560 × 1920	>5000 (monochromatic-red, green, blue)	^118^
	~5	5	N/A	5080 (monochromatic-red, green, blue)	^120^
	5	7.5	1080 × 780	3400 (green)	^121^
Growth integration	10	50	N/A	508 (red, green, blue)	^122^
	450	N/A	N/A	N/A	^124^
	50	80	20 × 20	N/A (blue)	^125^
	10	20	32 × 32	1270 (blue)	^127^
	0.22–0.63	~1	N/A	N/A (red, orange, green, blue)	^110^

**Fig. 5:**
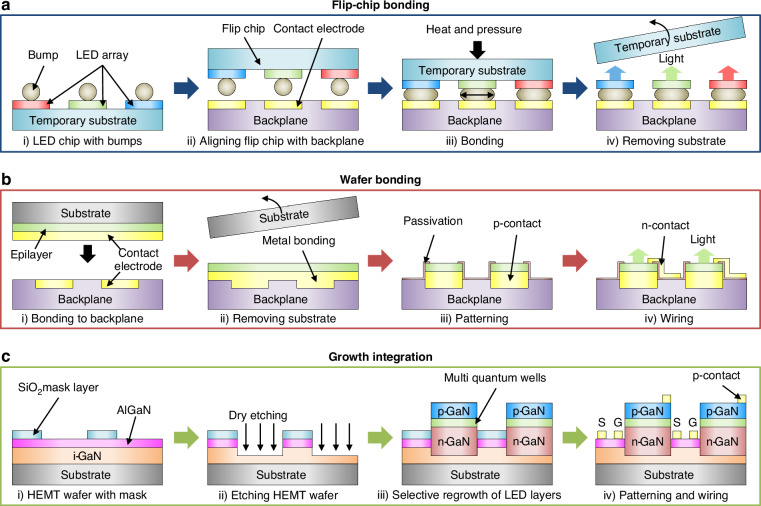
**Electrical interconnection techniques for micro-LED displays.** **a** Schematic illustration of flip-chip bonding process. **b** Schematic illustration of wafer bonding process. **c** Schematic illustration of growth integration

Flip-chip bonding, also known as solder bump bonding, is the method used for large-scale micro-LED displays due to the high throughput and low cost^113–115^. This technique is often referred to as “face-down” assembly and provides a repair-friendly configuration, as it avoids routing interconnects over the top of the LED chips. A typical process involves the mass transfer of RGB LED chips on a temporary substrate (i.e., carrier substrate) coated with a sacrificial layer, the formation of patterned bumps (indium or gold) using thermal evaporation or electroplating techniques, or the application of anisotropic conductive film (ACF, a composite material containing conductive particles embedded in a polymer matrix), alignment and thermal compression bonding between the chips and backplane, and removal of temporary substrate (Fig. 5a). One limitation of flip-chip bonding is that the large bump size (~10 µm) impedes manufacturing high-resolution displays. A recent study demonstrated reduction in bump size through a formic acid reflow process^116^, exposing indium bumps to acid at high temperature, but this can lead to misalignment between the chips and backplane due to the mismatch in thermal expansion coefficients. Another method is to fabricate micro-tubes filled with bumps on the backplane, which allows room-temperature bonding and can achieve a bump size of ~5 µm^117^.

Wafer bonding, also known as metal-metal bonding or eutectic bonding, is used to electrically interconnect micro-LEDs with a backplane without using solder bumps, which is necessary for manufacturing high-pixel-density micro-displays^118–120^. Figure 5b illustrates the process flow involving the deposition of Au-terminated metal layers on the LED wafer and backplane, metal-metal bonding at high temperature and pressure to covalently bond the two metal layers, and substrate removal via LLO or wet etching process^118,121^. Photolithography, reactive ion etching, passivation, and metallization processes complete the micro-LED fabrication. JBD used a wafer bonding process to demonstrate monochrome active-matrix micro-LED displays with pixel pitch of 4 μm and pixel density of 6350 ppi for AR displays^118,119^. More recently, JBD introduced monochromatic micro-LEDs with a pixel pitch of 2.5 μm using wafer bonding^105^. Existing procedures, however, are limited to monochrome displays, necessitating further research to realize full-color micro-LED micro-displays. This can be achieved through monolithic integration to create vertical full-color micro-LEDs or by using QD color conversion layers^122,123^, as discussed in previous sections.

Growth integration method achieves monolithic integration of micro-LEDs with TFTs via sequential growth of device layers on a single substrate, fabrication of pixels side-by-side, and formation of electrical interconnection between device pairs to obtain active-matrix micro-LED displays. This reduces the process complexity by eliminating the need for alignment and mass transfer processes. Recent reports demonstrate selective area growth of InGaN-based micro-LEDs on GaN/AlGaN high electron mobility transistor (HEMT) wafers using silicon dioxide (SiO_2_) masks, thereby achieving monochrome active-matrix micro-LED displays (Fig. 5c)^124,125^. To this end, low-temperature epitaxial growth below 450 °C is required, particularly allowing direct growth on metal pads without damaging underlying circuitry. Alternatively, high-temperature-resistant backplane technologies, such as AlGaN/GaN HEMT-based transistors, could be employed to withstand the thermal budget of epitaxial processes^126^. Another approach involves the low-temperature growth and fabrication of 2D materials-based TFTs on LED substrate^122,127^. Additional research will be needed, however, to improve pixel density, achieve full-color micro-LED integration, and improve stability and mass production of HEMT and 2D materials-based TFTs.

## Detection and repair of defective pixels

Detection and repair are essential in manufacturing micro-LED displays, which consist of extremely small and highly dense arrays of pixels. Pixels that have no emission (dead), irregular brightness (dim), unwanted color shifts, or are permanently on (stuck) can be identified via photoluminescence (PL), electroluminescence (EL), and machine learning-based analysis methods^128–130^. PL analysis involves optical excitation of the LED on the epitaxial wafer using an external laser or UV source, followed by spectral analysis of the emitted PL. Material quality is evaluated based on the PL wavelength and intensity, enabling the identification of defective micro-LED chips prior to the mass transfer process. EL analysis involves driving current through transferred micro-LEDs under actual display operating conditions and evaluating the spectral properties of emitted light. Lastly, machine learning has been used in conjunction with high-resolution cameras and microscopes to quickly scan the display for anomalies in brightness, color, and uniformity. Deep learning models are trained on defect patterns to classify pixel issues, allowing high-throughput and automated defect detection.

Broadly, defective micro-LEDs are repaired both pre-transfer and post-transfer. Pre-transfer repair involves the exclusion of defective pixels (identified via PL analysis) from mass transfer onto the backplane. Each pixel site on the LED epi-wafer consists of multiple spare micro-LEDs, of which only non-defective ones are transferred. Post-transfer repair involves the selective removal and replacement of defective micro-LEDs (identified via EL analysis) from the display backplane. The specific process includes removal of cured adhesive, elimination of all particles and contaminants, re-application of adhesive material, and transfer of non-defective pixels. Post-transfer repair is time-consuming and significantly increases the manufacturing cost of micro-LED displays, especially for higher-resolution displays.

## Emerging display applications

Recent years have seen the rise of various displays with novel functionalities compared to traditional flat-panel displays. Below, we discuss the mechanisms and materials/design requirements of emerging display applications such as transparent displays, deformable displays, and virtual and augmented reality (VR/AR) displays.

**Fig. 6:**
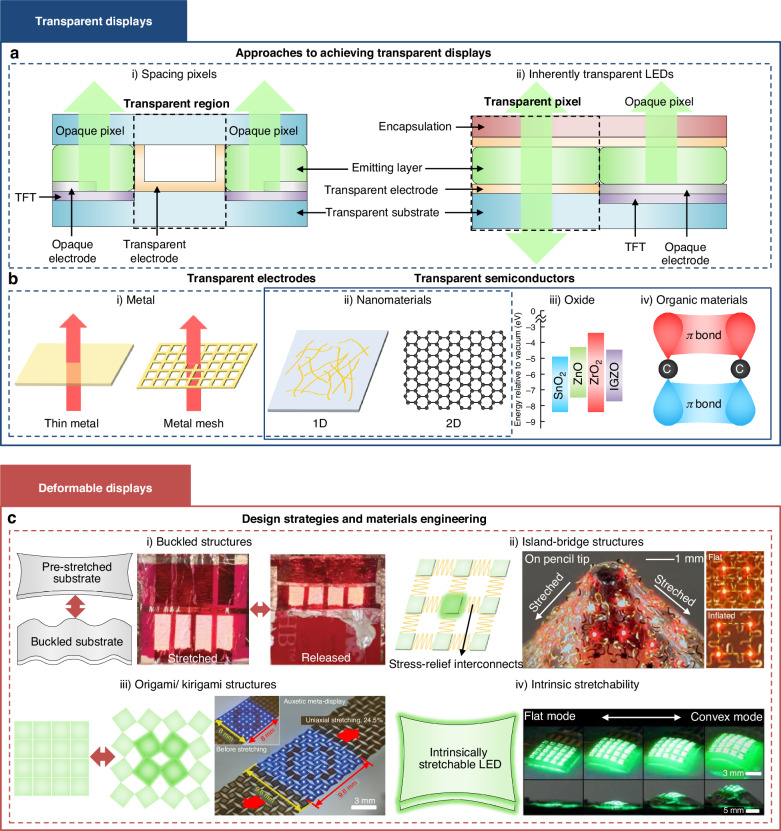
**Emerging display applications.** **a** Two approaches to achieving transparent displays. **b** Schematic illustration of transparent electrodes and semiconductors. **c** Design strategies and materials engineering of deformable displays. i) Reproduced with permission^143^. Copyright 2013, Springer Nature. ii) Reproduced with permission^84^. Copyright 2010, Springer Nature. iii) Reproduced under the terms of the Creative Commons CC-BY-NC-ND license^144^. Copyright 2022, John Wiley and Sons. iv) Reproduced with permission^145^. Copyright 2024, Springer Nature

Transparent displays, also known as see-through displays, have recently drawn interest from the industry due to their envisioned applications in shop windows, product showcases, heads-up displays, and home appliances, which allow quality-of-life improvements. Several companies have recently introduced prototype displays based on micro-LEDs and OLEDs at exhibitions such as the Consumer Electronics Show (CES) 2024. To achieve transparency, display components such as emitters, electrodes, and driving circuits must be carefully engineered. One approach is to fabricate LEDs in a grid layout on a transparent substrate, where the spaces between opaque grid structures are transparent, allowing the display to appear semi-transparent at sufficient viewing distances (Fig. 6a (i))^131^. This is necessary as conventional III–V/III–N micro-LEDs and phosphorescent/TADF OLEDs are opaque. Micro-LEDs do offer clear advantages over OLEDs due to their higher luminance and ultra-small chip size, which enable both high visibility (especially for usage outdoors or under strong ambient light intensities) and transparency^132^. The grid pattern must also be designed to maximize transparency and image quality. Another approach is to use inherently transparent LEDs such as OLEDs and QD-LEDs, which allows the display to become transparent when turned off (Fig. 6a (ii))^133,134^. The current limitation is that the thick encapsulation layers used to prevent oxygen and moisture reduce transparency. Advances in encapsulation layers, antireflection coatings, microlenses, and optimized pixel geometries could enhance light extraction efficiency and the perceived transparency of the display^135^.

The overall display transparency also relies on that of the electrodes and semiconductors used in TFTs. Indium tin oxide (ITO) is the most widely used for transparent electrode due to its high optical transmittance (~90%) and low sheet resistance (~10 Ω/□), but it is limited by brittleness and the high cost of fabrication. Alternatives such as ultrathin metals, mesh-patterned electrodes, and nanomaterials such as silver nanowires (AgNWs), carbon nanotubes (CNTs), and graphene offer flexibility and high transparency but suffer from issues such as high surface roughness and poor stability (Fig. 6b (i, ii))^136,137^. Transparent semiconductors can be categorized into oxide, organic, and nanomaterial-based semiconductors (Fig. 6b (ii, iii))^138–141^. Oxide semiconductors such as IGZO provide high transmittance and moderate electron mobility but require encapsulation layers to prevent degradation. Organic semiconductors offer flexibility and solution processability but are limited by low mobility and stability issues. Nanomaterial-based semiconductors, including graphene and transition metal dichalcogenides (TMDs), exhibit excellent mobility and transparency but face challenges related to contact resistance, processing difficulties, and scalability, limiting their practical implementation.

Deformable displays, including foldable, rollable, flexible, and stretchable devices, have potential applications in wearable displays, curved displays, and portable displays that can provide quality-of-life improvements in a wide range of areas, including fitness tracking, fashion, and entertainment^142^. Deformable displays can be obtained by using (i) structural designs that enable mechanical flexibility/stretchability, or (ii) intrinsically stretchable materials. Examples of mechanical designs that allow deformability include buckled structures formed on pre-strained elastomer substrates, island-bridge designs with stretchable metal interconnects, and origami or kirigami designs (Fig. 6c (i–iii))^84,143,144^. These engineered stress-relief designs allow the brittle components of the display to undergo repeated deformation without breaking. Intrinsically stretchable displays use light-emitting nanomaterials (OLEDs, QDs) embedded in elastomer matrix, conductive polymers, and liquid metals, to enable systems that can stretch uniformly upon applied stress (Fig. 6c (iv))^145,146^. This approach has the advantage of allowing higher pixel density by eliminating the need for engineered stress-relief structures, which occupy considerable space. However, their current limitations include low stability/electrical performance compared to rigid displays, a limited selection of materials, and low resolution and yield.

**Fig. 7:**
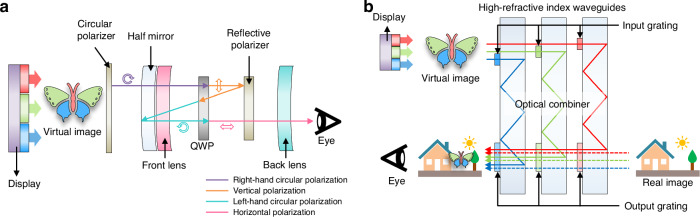
**VR/AR displays.** **a** Structure of VR displays. **b** Structure of AR displays with waveguides

VR displays are near-eye displays that create a simulated 3D environment by presenting each eye with a slightly different image, allowing the users to perceive depth and feel immersed in a virtual world they can interact with in real-time. VR displays can make a transformative impact in education and training (medical/military operations), entertainment (gaming, film, live events), and healthcare (remote surgery). In a standard VR system, the light emitted by a display undergoes multiple polarization and reflection processes to enhance image clarity, minimize distortion, and ensure a wide field of view, which helps project the flat 2D images into our eyes as focused 3D images (Fig. 7a)^147–149^. Due to the small viewing distance (1–2 inches), VR displays need to have ultra-high-pixel density to reduce the noticeable gaps between pixels, known as the screen-door effect, which prevents immersion. LCDs were used in early commercialized products such as Meta Quest, while the latest VR device, Apple Vision Pro, uses micro-OLEDs, which offer lighter weight and higher pixel density (3386 ppi). VR displays require moderate brightness, as the headsets block ambient light. Current research focuses on increasing the pixel density, reducing power consumption, and minimizing the size and weight of VR displays. Significant efforts are being made to address technical challenges in fabricating FMMs, including improving their durability, enabling high-resolution patterning, and ensuring the deposition uniformity of OLED materials within a single pattern. The key characteristics of different commercial VR display products are summarized in Table 3.

**Table 3:** Benchmark Table comparing key parameters of commercial VR/AR display products

VR/AR displays	Form factor	Optic engine	Resolution	ppi	Transparency (%)		Weight (g)
Meta Quest 3	VR headset	LCD	2064 × 2208	1218	Blocked	515	
Apple Vision Pro	VR headset	Micro-OLED	3660 × 3200	3386	Blocked	600–650	
Sony PSVR 2	VR headset	OLED	2000 × 2040	800	Blocked	560	
Meta Quest Pro	VR headset	QD-LCD	1800 × 1920	1059	Blocked	722	
Microsoft Hololens 2	AR glasses	Laser	1440 × 936	N/A	~30	566	
Xreal Air 2 Pro	AR glasses	Micro-OLED	1920 × 1080	4032	20–30	75	
Vuzix Blade 2	AR glasses	LCD	480 × 853	N/A	20–30	90	
Oppo Air glasses 3	AR glasses	Micro-LED	~4096 × 2160	N/A	>40	50	
Meta Orion	AR glasses	Micro-LED	~720 × 540	N/A	60–70	98	

AR displays are near-eye displays that overlay digital information such as images, text, or 3D models on top of the real world, enabling users to see both the physical environment and the added digital content simultaneously. Their main benefit includes providing improved awareness and decision-making for the user, which can be useful for a wide range of activities, including travel, design, manufacturing, medical practice, and military operations. For practical use, AR displays require highly immersive, glasses-type displays with a minimum resolution of 5000 ppi (or ~60 pixels per degree (ppd)), a viewing angle of at least 60°, a weight of 40 grams or less, and slim form factors. Micro-displays based on liquid crystal on silicon (LCoS)^150^ and laser beam scanning (LBS)^151^ technologies were traditionally used for AR displays^22^ (for example, Microsoft HoloLens was based on LBS; refer to Table 3).

In contrast, the latest AR glasses based on JBD Hummingbird micro-displays, such as Meta Orion, use three monochrome micro-LED displays (for RGB) which deliver full-color images to the eye with the help of optical combiners (waveguides, e.g.) and focal plane directors (Fig. 7b)^152^. Each monochrome image is coupled into a high-refractive index waveguide via input gratings, undergoes multiple internal reflections, and exits through output gratings that spatially expand and deliver the combined polychrome image to the user’s eye. A major drawback of this design is the significant optical loss. Because LEDs emit light with a Lambertian angular distribution, only a small fraction of the emitted light enters the waveguide, and even less reaches the eye after multiple reflections. To mitigate this, micro-lens arrays (MLAs) are used to satisfy the étendue requirements and enhance light coupling efficiency; however, their performance has practical limits. Additional losses occur at optical combiners and during the projection of images across multiple focal planes. The total loss for the AR glasses is known to be more than 99%. Consequently, AR displays require extremely high luminance for outdoor visibility^102,153^, which makes micro-LEDs (>10^6^ nits) more viable than micro-OLEDs (~10^3^ nits). Moreover, power consumption is also critical for wearable AR displays. Qian et al. recently presented a comprehensive comparison of five microdisplay technologies (micro-LED, OLED, LCoS, DLP, and LBS) and identified micro-LED and advanced LCoS as the most promising candidates for low-power AR glasses^154^. While LCoS currently offers a more mature solution, micro-LEDs are widely considered the most theoretically ideal technology for next-generation AR glasses, assuming that key fabrication and integration hurdles are addressed. Designing front-end optics capable of producing collimated emission from LEDs remains a key challenge for high-efficiency AR glasses. Additional research is also needed to improve the brightness, resolution, and field of view, while reducing the weight and size of the system.

## Summary and outlook

The display technology has made remarkable strides in recent years, achieving significant improvement in image quality, reduction in size and weight, and discovery of novel functional applications, such as transparency, deformability, and immersion. While OLEDs remain the dominant commercial display platform, micro-LEDs are emerging as the next-generation solution due to their superior brightness, efficiency, long lifetime, and scalability. However, widespread adoption of micro-LEDs still faces several critical challenges across materials, process integration, and system-level design. Mass transfer and flip-chip bonding methods are expected to be used primarily for medium- to low-resolution flat-panel displays, while continued efforts will be made to improve transfer yield, alignment accuracy, and cost efficiency. For high-resolution applications such as AR/VR micro-displays, monolithic integration and wafer bonding are promising approaches to achieve ultra-high-ppi and full-color RGB integration. Other emerging strategies, including monolithic growth, tunnel junctions, 2D-material-assisted layer transfer, and selective full-color nanowire growth, have shown potential to overcome existing limitations in device stacking, color control, and pixel miniaturization. In transparent displays and deformable displays, micro-LEDs offer advantages in reduced pixel size as well as enhanced transparency and flexibility, although optical loss and system-level complexity remain issues to address. Particularly for AR systems, high luminance requirements (>10^6^ nits), etendue constraints (emission angle within ±5°), and waveguide coupling efficiency pose major challenges, necessitating novel optical architectures such as micro-lens arrays, collimated emitters, and multi-focal plane projection optics. Looking forward, advances in epitaxial growth, chip-scale RGB vertical stack packaging, bonding technologies, and integrated backplane electronics—including HEMTs and 2D-TFTs—will be challenging to realize manufacturable and scalable micro-LED displays. With continued innovation, micro-LEDs are poised to revolutionize next-generation displays across consumer electronics, wearable systems, automotive displays, and beyond, potentially redefining how we interact with information toward “metaverse era”—just as smartphones had done for the previous decade.
